# Kinetics of HLA antibodies: A Titanic effort is required

**DOI:** 10.12669/pjms.296.3701

**Published:** 2013

**Authors:** Younis Skaik

Recently, Jackman et al., have published a very interesting paper entitled ´Low-level HLA antibodies do not predict platelet transfusion failure in TRAP study participants.^1^ The authors have profoundly explained the possible mechanisms that might be responsible for the platelet refractoriness in the 19% of recipients who had no detectable human leukocyte antigens (HLA) antibodies using the lymphocytoxocity assay (LCA), but were at low levels detected using the sensitive bead-based multiplexing assay. A galaxy of old studies have shown and attributed the low HLA detectable antibody levels in the acute myeloid leukemia (AML) patients to the fluctuation kinetics of the HLA antibodies over time,^2^ or more importantly to the what called “immune tolerant”.^3^ That tolerance state can be produced by the exposure of the AML patients to large amounts of the HLA antigens from the multiple transfusions as shown in vitro and in the murine models.^4^^-^^8^ Another possible way of the persistent exposure to the HLA molecules in the AML patients and hence the possibility of the immune tolerance is the transfer of the HLA molecules from the transfused platelets to the leukemic cells, which is a process known as trogocytosis, and the growing evidence suggests its role in the tumor immunology.^9^ In conclusion, the failure of an assay for the HLA antibodies detection does not mean necessarily that the unsuitability of the assay, but one should keep in mind the titanic fluctuations of the HLA antibodies over time and the interplay between the persistent exposure to the HLA molecules and the modulation of the immune response. 
